# Accuracy of high-risk HPV DNA PCR, p16^(INK4a)^ immunohistochemistry or the combination of both to diagnose HPV-driven oropharyngeal cancer

**DOI:** 10.1186/s12879-022-07654-2

**Published:** 2022-08-06

**Authors:** Cindy Simoens, Tarik Gheit, Ruediger Ridder, Ivana Gorbaslieva, Dana Holzinger, Eric Lucas, Susanne Rehm, Peter Vermeulen, Martin Lammens, Olivier M. Vanderveken, Rekha Vijay Kumar, Nitin Gangane, Alessandro Caniglia, Fausto Maffini, Maria Belén Lloveras Rubio, Devasena Anantharaman, Susanna Chiocca, Paul Brennan, Madhavan Radhakrishna Pillai, Rengaswamy Sankaranarayanan, Johannes Bogers, Michael Pawlita, Massimo Tommasino, Marc Arbyn, the HPV-AHEAD study group, Christine Carreira, Sandrine McKay-Chopin, Rudrapatna S. Jayshree, Kortikere S. Sabitha, Ashok M. Shenoy, Alfredo Zito, Fausto Chiesa, Marta Tagliabue, Mohssen Ansarin, Subha Sankaran, Christel Herold-Mende, Gerhard Dyckhoff, George Mosialos, Heiner Boeing, Xavier Castellsagué, Silvia de Sanjosé, Marisa Mena, Francesc Xavier Bosch, Laia Alemany, Pulikottil Okkuru Esmy, Manavalan Vijayakumar, Aruna S. Chiwate, Ranjit V. Thorat, Girish G. Hublikar, Shashikant S. Lakshetti, Bhagwan M. Nene, Amal Ch. Kataki, Ashok Kumar Das, Kunnambath Ramadas, Thara Somanathan

**Affiliations:** 1 Unit of Cancer Epidemiology, Belgian Cancer Centre, Sciensano, Juliette Wytsmanstraat 14, 1050 Brussels, Belgium; 2 AMBIOR, Laboratory for Cell Biology & Histology, University of Antwerp, Antwerp, Belgium; 3 Early Detection, Prevention and Infections Branch, International Agency for Research on Cancer (IARC), Lyon, France; 4 Roche Diagnostics GmbH, Mannheim, Germany; 5 Ventana Medical Systems, Inc. (Roche Diagnostics Solutions), Tucson, AZ USA; 6 Infections and Cancer Epidemiology, Research Program Infection, Inflammation and Cancer, Deutsches Krebsforschungszentrum, Heidelberg, Germany; 7 Laboratory for Pathological Anatomy, Sint Augustinus Hospital, GZA, Antwerp, Belgium; 8 Department of Pathology, Antwerp University Hospital, Edegem, Belgium; 9 Center for Oncological Research, Faculty of Medicine and Health Sciences, University of Antwerp, Antwerp, Belgium; 10 Department of Otorhinolaryngology, Head and Neck Surgery, Antwerp University Hospital, Edegem, Belgium; 11 Department of Translational Neurosciences, Faculty of Medicine and Health Sciences, University of Antwerp, Antwerp, Belgium; 12 Kidwai Memorial Institute of Oncology, Bangalore, Karnataka 560029 India; 13 Mahatma Gandhi Institute of Medical Sciences, Sevagram, Wardha, Maharashtra State 442102 India; 14 IRCCS Istituto Tumori “Giovanni Paolo II”, Bari, Italy; 15 Division of Pathology, IEO, European Institute of Oncology IRCCS, Milan, Italy; 16 Hospital del Mar, Parc de Salut Mar, Pg/Marítim 25-29, 08003 Barcelona, Spain; 17 Rajiv Gandhi Centre for Biotechnology, Poojappura, Thiruvananthapuram, Kerala 695014 India; 18 Department of Experimental Oncology, IEO, European Institute of Oncology IRCCS, Milan, Italy; 19 Genomic Epidemiology Branch, International Agency for Research On Cancer (IARC), Lyon, France; 20 Research Triangle Institute (RTI) International India, New Delhi, India

**Keywords:** HPV DNA PCR, p16^(INK4a)^ IHC, HPV mRNA, Oropharyngeal carcinoma, HPV-AHEAD

## Abstract

**Background:**

The incidence of high-risk human papillomavirus (hrHPV)-driven head and neck squamous cell carcinoma, in particular oropharyngeal cancers (OPC), is increasing in high-resource countries. Patients with HPV-induced cancer respond better to treatment and consequently have lower case-fatality rates than patients with HPV-unrelated OPC. These considerations highlight the importance of reliable and accurate markers to diagnose truly HPV-induced OPC.

**Methods:**

The accuracy of three possible test strategies, i.e. (a) hrHPV DNA PCR (DNA), (b) p16^(INK4a)^ immunohistochemistry (IHC) (p16), and (c) the combination of both tests (considering joint DNA and p16 positivity as positivity criterion), was analysed in tissue samples from 99 Belgian OPC patients enrolled in the HPV-AHEAD study. Presence of HPV E6*I mRNA (mRNA) was considered as the reference, indicating HPV etiology.

**Results:**

Ninety-nine OPC patients were included, for which the positivity rates were 36.4%, 34.0% and 28.9% for DNA, p16 and mRNA, respectively. Ninety-five OPC patients had valid test results for all three tests (DNA, p16 and mRNA). Using mRNA status as the reference, DNA testing showed 100% (28/28) sensitivity, and 92.5% (62/67) specificity for the detection of HPV-driven cancer. p16 was 96.4% (27/28) sensitive and equally specific (92.5%; 62/67). The sensitivity and specificity of combined p16 + DNA testing was 96.4% (27/28) and 97.0% (65/67), respectively. In this series, p16 alone and combined p16 + DNA missed 1 in 28 HPV driven cancers, but p16 alone misclassified 5 in 67 non-HPV driven as positive, whereas combined testing would misclassify only 2 in 67.

**Conclusions:**

Single hrHPV DNA PCR and p16^(INK4a)^ IHC are highly sensitive but less specific than using combined testing to diagnose HPV-driven OPC patients. Disease prognostication can be encouraged based on this combined test result.

**Supplementary Information:**

The online version contains supplementary material available at 10.1186/s12879-022-07654-2.

## Background

In high-resource countries, human papillomavirus (HPV)-driven head and neck squamous cell carcinoma represents an increasing health problem, particularly in cancers of the oropharyngeal region, including base of tongue and tonsils [1–4]. An estimated 20–40% of oropharyngeal cancers (OPC) is believed to be caused by HPV infection, and the large majority of them (> 80%) are due to HPV16 [2, 5]. Patients with HPV-induced cancers respond better to treatment and consequently have better survival than patients with HPV-unrelated OPC [6–11]. HPV-positive tumours, compared to the HPV-negative ones, are characterized by multiple molecular and clinic-pathological differences [12], which should be further investigated. These considerations highlight the importance of reliable and accurate markers, or marker combinations, to diagnose truly HPV-induced OPC and guiding patients’ risk-stratification.

The most widely applied detection method was based on PCR amplification of viral DNA to determine HPV-positivity. However, several independent studies have highlighted that PCR-based assays for the detection of HPV DNA are not sufficiently accurate to establish the viral causality [13–17]. These PCR-based methods are highly sensitive and can detect even a few DNA copies per sample, which might yield false-positive results mainly reflecting transient infections [18–20]. Additional markers, such as the presence of viral E6/E7 mRNA transcripts and p16^(INK4a)^ expression as surrogates for HPV-induced transformation, allow a more accurate classification of HPV-driven head and neck cancers (HNC) [15, 21–25].

Nowadays, HPV E6/E7 oncogene transcript detection is considered the gold standard, for HPV-induced malignancies particularly depend on the carcinogenic potential of the HPV E6 and E7 oncoproteins [23, 26]. Nevertheless, viral transcript detection is laborious and may not be feasible everywhere in daily lab routine. This is particularly true for the detection of mRNA transcripts in formalin-fixed, paraffin-embedded (FFPE) tissue specimens, which are commonly used during routine diagnostic work up; however, RNA integrity may be affected by the fixation protocol.

HPV E7 oncogenic signaling brings about substantial overexpression of the cellular protein p16^(INK4a)^ in HPV-transformed cells [27]. Therefore, the immunohistochemical detection of p16^(INK4a)^ overexpression is presently applied as a surrogate biomarker of HPV-transformed cervical epithelium [28–30]. Similarly, p16^(INK4a)^ immunohistochemistry (IHC) is regularly used to establish HPV association in HNC [22, 23, 31–33]. Apart from single p16^(INK4a)^ IHC, combined p16^(INK4a)^ and HPV DNA detection by PCR is oftenly used.

### HPV-AHEAD (FP7 funded network)

The HPV-AHEAD study (“Role of human papillomavirus infection and other co-factors in the aetiology of head and neck cancer in Europe and India”) group comprised partners from six European countries and from India (website HPV-AHEAD: https://hpv-ahead.iarc.fr/). The main goal of the study was to perform a comprehensive analysis on a large number of HNC cases to clarify pathogenic pathways in HNC carcinogenesis, and to identify clinically useful biomarkers.

In a previous publication [34], the results of the histological and molecular assessment of 1039 archived HNC specimens from Belgian patients were described, primarily using the detection of HPV DNA, mRNA, and p16^(INK4a)^ IHC. In this study, we focus on the accuracy of these tests—individually and in combination—to diagnose hrHPV-driven oropharyngeal cancer*.*

## Methods

### Patient selection, clinical information and tissue specimen collection for the Belgian cohort

The overall study included patients with oropharyngeal, oral cavity, laryngeal, hypopharyngeal, and unspecified head and neck squamous cell carcinoma. For a detailed description of the patient characteristics, we refer to the previous publication of the Belgian HPV-AHEAD study data [34]. For the analyses in this study, the focus was on the oropharyngeal cancers [ICD-O-3 codes: C01 (base of tongue); C02.4 (lingual tonsil); C05.1 (soft palate); C05.2 (uvula); C09 (tonsil): C09.0, C09.1, C09.8 and C09.9; C10 (oropharynx): C10.0–10.4, C10.8 and C10.9].

FFPE tumour blocks and clinical information were collected from OPC patients treated in two Belgian hospitals (GZA and UZA), diagnosed between 1980 and 2010.

Ethical clearance was obtained from the Ethical Committees of ‘GZA hospitals’ (ref nb: BVDE/hp/2013/01.147), ‘UZA & UA (University of Antwerp)’ (ref nb: 11/47/362) and IARC, Lyon, France (ref nb: 11–30).

### Preparation of tissue sections (University of Antwerp, Antwerp, Belgium)

FFPE-tissue blocks were all processed at the UA laboratory of cell biology and histology, following the optimized HPV-AHEAD sectioning protocol [34, 35]. Briefly, minimal ten sections (S) were prepared from each FFPE block. The first (S1) and the last (S10) 5 µm sections were hematoxylin and eosin (H&E) stained for morphologic histology interpretation and used to check for the presence of tumour. S2 and S9 (5 µm) were used for p16^(INK4a)^ IHC staining, while the 10 µm sections S3–5 and S6–8 were used for the extraction of RNA and DNA, respectively.

### Histological review

All sections were re-evaluated by the HPV-AHEAD pathology review panel. The review was blinded with respect to the original local diagnosis. Only FFPE blocks where S1 and S10 H&E sections reflected squamous tumour tissue were included in the analysis [34].

### HPV E6*I mRNA analysis (DKFZ, Heidelberg, Germany)

All OPC cases were analysed for the presence of: (i) HPV16 E6*I mRNA, and (ii) ubiquitin C (ubC) mRNA as a cellular mRNA positive control (housekeeping gene used for RNA quality control). OPC cases positive for DNA of a non-HPV16 genotype were additionally analysed for E6*I mRNA of the respective genotype. Specimens that were HPV E6*I and/or ubC mRNA-positive (RNA+) were considered RNA valid. Reverse Transcription-PCR was carried out using the QuantiTect Virus Kit (Qiagen, Hilden, Germany), as described in full in former HPV-AHEAD publications [34, 36]. The type-specific E6*I mRNA assays identifying transcripts of fourteen high-risk and six possible/probable high-risk HPV genotypes [37] were applied.

### HPV DNA genotyping (IARC, Lyon, France)

HPV DNA genotypes were detected by a E7 type-specific multiplex genotyping (E7-MPG) assay, which combines multiplex PCR and bead-based Luminex technology (Luminex Corporation, Austin, TX), as previously described [38, 39]. Type specific-MPG uses HPV type-specific primers targeting the E7 region of thirteen high-risk and six possible/probable high-risk HPV genotypes (HPV 16, 18, 26, 31, 33, 35, 39, 45, 51, 52, 53, 56, 58, 59, 66, 68a and b, 70, 73 and 82), as well as two low-risk HPV genotypes (HPV 6 and 11). Two primers for amplification of the beta-globin (β-globin) gene were also included to control for the DNA quality of each specimen. A detailed description of the DNA extraction and further HPV genotyping test characteristics can be found in the previous publication on the Belgian HPV-AHEAD study [34]. Genotyping controls and DNA preparation were blindly analysed, and no sign of contamination of negative controls was detected during the laboratory work.

### p16^(INK4a)^ expression (Roche mtm laboratories, Mannheim, Germany)

Expression of p16^(INK4a)^ was evaluated by IHC, with a dual-immunostaining protocol for the simultaneous immunostaining of both p16^(INK4a)^ and Ki-67 biomarkers (CINtec PLUS kit, Roche mtm laboratories AG, Mannheim, Germany), as previously described in the HPV-AHEAD publications [34, 36]. As in all HPV-AHEAD studies [34, 36, 40], a continuous, diffuse staining for p16^(INK4a)^ within the cancer area of the tissue sections was considered as positive, while a focal staining or no staining was considered negative. IHC slides were analysed without knowledge of any other clinical information (including HPV DNA and RNA status) by the scientists RR or DH and reviewed by one of the European members of the HPV-AHEAD pathology review panel (JPB, BLR, or FM). Discrepant cases were re-evaluated by a pathologist outside the review panel (AC), and final classification of the staining was based on majority consensus results.

Corresponding to the former Belgian HPV-AHEAD publication [34], the p16 slides with technical issues were restained and re-evaluated, to minimize the number of missing results.

### Statistical analysis

Presence of viral mRNA was considered as the reference indicating HPV etiology. The accuracy of three possible test strategies was analysed: (a) hrHPV DNA PCR alone, (b) p16^(INK4a)^ IHC alone, and (c) the combination of p16^(INK4a)^ IHC and hrHPV DNA PCR; where positivity is defined by a co-positive result with both tests, a negative result by a co-negative result for both tests, and a discordant result by only one test being positive and the other being negative. Test strategy (c) involves three algorithms: ALGORITHM 1 (p16 + DNA) consists of p16^(INK4a)^ IHC and hrHPV DNA PCR on all samples; ALGORITHM 2 (p16 → DNA) involves p16^(INK4a)^ staining of all samples, followed by the HPV DNA test only on the p16^(INK4a)^-positive samples; ALGORITHM 3 (DNA → p16) involves HPV DNA PCR on all samples, followed by p16^(INK4a)^ staining only on the HPV DNA-positive samples.

Sensitivity was defined as the proportion of test positive samples among HPV RNA-positive patients. Specificity was defined as the proportion of negative test results among HPV RNA-negative patients. Relative sensitivity and specificity of each test compared to the other tests were also assessed. Binomial 95% confidence intervals (95% CIs) were computed for proportions. Given the matched testing of the same patient specimens, McNemar 95% CIs were computed for relative accuracy parameters. Continuous variables were summarized by their mean and 95% CIs. ANOVA was used to compare mean age by gender.

Statistical analyses were performed using STATA 16 (StataCorp, College Station, TX). p-values were two sided and statistical significance was set at p equal or less than 0.05.

## Results

### Study population characteristics

Ninety-nine of 116 OPC patients were included in the analysis, after exclusions based on the pathology review (for reasons of not being a squamous cell carcinoma or not reflecting invasive cancer, N = 13) and beta-globin PCR negativity (N = 4). 71.7% (71/99) of the patients were male, 26.3% (26/99) were female, and for the remaining two samples the gender of the patients was not specified. The mean age at diagnosis was 67.3 years (95% CI 64.9–69.7) and was not different between males and females (p = 0.47).

### HPV prevalence in OPC cases

HPV prevalence was determined by HPV DNA PCR, p16^(INK4a)^ IHC, E6*I mRNA, and all combinations of tests in the HPV-AHEAD study. Out of 99 OPC cases, 36.4% (36/99) contained HPV DNA, 34.0% (33/97) showed p16^(INK4a)^ positivity, and 28.9% (28/97) were HPV RNA-positive (Table 1). Twenty-six of the 28 HPV RNA-positive samples were positive for HPV16 (92.9%), the remaining 2 samples contained HPV18 (7.1%).

**Table 1:** Prevalence of HPV determined by HPV DNA PCR (DNA), p16^(INK4a)^ IHC (p16), E6*I mRNA (RNA), and all combinations of tests, in oropharyngeal cancer

Marker positivity		Oropharyngeal cancer		
		Positive	Proportion	(95% CI)
DNA	(N = 99)	36^a^	36.4%	(27.4–46.4)
p16	(N = 97)^b^	33	34.0%	(25.2–44.1)
RNA	(N = 97)^c^	28	28.9%	(20.6–38.8)
p16 + DNA	(N = 97)	30	30.9%	(22.5–40.9)
DNA + RNA	(N = 97)	28	28.9%	(20.6–38.8)
RNA + p16	(N = 95)	27	28.4%	(20.2–38.4)
p16 + DNA + RNA	(N = 95)	27	28.4%	(20.2–38.4)

^a^1 lrHPV (HPV6) and 35 hrHPV

^b^2 equivocal results after final re-review

^c^2 samples gave an invalid RNA test result

The proportion of HPV-positive OPCs varied between 28.4% (27/95) and 28.9% (28/97) for all combinations of tests that included RNA testing (DNA + RNA, p16 + RNA, DNA + p16 + RNA). For the combination of testing for HPV DNA and p16^(INK4a)^ IHC, the overall prevalence was slightly higher (30.9%, 30/97).

### Absolute and relative accuracy in the oropharynx and its subsites

Both absolute and relative accuracy of hrHPV DNA detection by PCR (hrHPV DNA), p16^(INK4a)^ IHC (p16), and combined p16^(INK4a)^ and DNA testing algorithms (p16 + DNA, p16 → DNA and DNA → p16), to identify a transforming HPV infection were calculated. In the following tables, only the results are shown for the samples that had valid results on all three tests performed (N = 95).

All RNA-positive OPC cases were positive for hrHPV DNA (100%, 28/28), in contrast to one out of 28 HPV RNA-positive samples that did not have a positive p16^(INK4a)^ result (3.6%). Five out of 67 RNA-negative cases showed hrHPV DNA-positivity (7.5%), the same as observed for p16^(INK4a)^ IHC. This number would be further lowered to only 2 positive cases out of 67 RNA-negatives (3.0%), if hrHPV DNA and p16^(INK4a)^ testing (p16 + DNA) were combined. The same holds for the other algorithms where all samples would initially be tested on p16^(INK4a)^ (p16 → DNA) or hrHPV DNA (DNA → p16), followed by the other test if the initial test result turned out positive. Data are detailed in Table 2, for all OPC cases, and for the tonsils and the base of tongue in specific. As the number of samples from other oropharyngeal subsites was very low, no relevant data can be shown.

**Table 2:** hrHPV DNA, p16^(INK4a)^ (p16) and combined hrHPV DNA/p16^(INK4a)^ test results by hrHPV RNA status in cancers of the oropharynx, tonsils and base of the tongue

		hrHPV RNA		
		Pos	Neg	
		N	N	Total
hrHPV DNA				
Oropharynx	Pos	28	5	33
	Neg	0	62	62
	Total	28	67	95
Tonsil	Pos	19	3	22
	Neg	0	20	20
	Total	19	23	42
Base of tongue	Pos	7	0	7
	Neg	0	14	14
	Total	7	14	21
p16				
Oropharynx	Pos	27	5	32
	Neg	1	62	63
	Total	28	67	95
Tonsil	Pos	18	2	20
	Neg	1	21	22
	Total	19	23	42
Base of tongue	Pos	7	2	9
	Neg	0	12	12
	Total	7	14	21
p16 + DNA				
Oropharynx	Pos	27	2	29
	Neg	1	65	66
	Total	28	67	95
Tonsil	Pos	18	2	20
	Neg	1	21	22
	Total	19	23	42
Base of tongue	Pos	7	0	7
	Neg	0	14	14
	Total	7	14	21
p16 → DNA				
Oropharynx	Pos	27	2	29
	Neg	1	65	66
	Total	28	67	95
Tonsil	Pos	18	2	20
	Neg	1	21	22
	Total	19	23	42
Base of tongue	Pos	7	0	7
	Neg	0	14	14
	Total	7	14	21
DNA → p16				
Oropharynx	Pos	27	2	29
	Neg	1	65	66
	Total	28	67	95
Tonsil	Pos	18	2	20
	Neg	1	21	22
	Total	19	23	42
Base of tongue	Pos	7	0	7
	Neg	0	14	14
	Total	7	14	21

The accuracy of hrHPV DNA PCR and p16^(INK4a)^ IHC to detect a transforming HPV infection in OPCs was calculated (Table 3). Highest sensitivity (100.0%, 28/28) was noted for hrHPV DNA testing, while specificity was equal for both hrHPV DNA and p16^(INK4a)^ (92.5%, 62/67). The best compromise was found in the combined testing of hrHPV DNA PCR and p16^(INK4a)^ staining, with 96.4% (27/28) sensitivity and 97.0% (65/67) specificity. When looking in more detail to the proposed algorithms, all provided equal accuracy, however, with a 60% reduction in the number of HPV DNA PCR or p16^(INK4a)^ IHC tests needed in the sequential testing algorithms.

**Table 3:** Absolute sensitivity and specificity of hrHPV DNA PCR (hrHPV DNA), p16^(INK4a)^ IHC (p16) and the combined testing (p16 + DNA) to identify a HPV-driven oropharyngeal cancer

Absolute	Sensitivity	Specificity
hrHPV DNA		
n/N	28/28	62/67
%	100%	92.5%
(95% CI)	(87.7–100)	(83.4–97.5)
p16		
n/N	27/28	62/67
%	96.4%	92.5%
(95% CI)	(81.7–99.9)	(83.4–97.5)
p16 + DNA		
n/N	27/28	65/67
%	96.4%	97.0%
(95% CI)	(81.7–99.9)	(89.9–99.6)

For the subgroup of the tonsils, p16(INK4a) staining alone was as sensitive (94.7%, 18/19) and specific (91.3%, 21/23) as the combined testing, in contrast to the base of tongue group, where all tests were 100% sensitive and specific, except for the p16(INK4a) specificity (85.7%, 12/14).

hrHPV DNA PCR testing was slightly more sensitive compared to p16^(INK4a)^ IHC (ratio: 1.04, 95% CI 0.97–1.11), but equally specific (ratio: 1.00, 95% CI not computable) (see Table 4 and Additional file 1: Table S1). The combination of hrHPV DNA and p16^(INK4a)^ was slightly more specific (ratio: 1.05, 95% CI 0.97–1.21) compared to both single tests.

**Table 4:** Relative sensitivity and specificity of hrHPV DNA PCR (DNA) vs p16^(INK4a)^ IHC (p16), and of DNA and p16^(INK4a)^ vs combined testing (p16 + DNA), to identify a HPV-driven oropharyngeal cancer

Relative	Sensitivity	Specificity
hrHPV DNA vs p16	1.04	1.00
(95% CI)	(0.97–1.11)	(1.00–1.00)
p16 + DNA vs p16	1.00	1.05
(95% CI)	(1.00–1.00)	(0.87–1.26)
p16 + DNA vs hrHPV DNA	0.96	1.05
(95% CI)	(0.90–1.04)	(0.87–1.26)

## Discussion

In these Belgian OPC patients, PCR testing for HPV DNA showed 36% positive cases. This proportion lies somewhere between other published results from different parts of the world. Castellsagué et al. found a considerably lower prevalence of HPV DNA: 24.9% in OPC, in a large international series of 3,680 HNC biopsies [33]. A very similar proportion of HPV DNA-positive oropharyngeal squamous cell carcinoma of 35.6% was found by Kreimer et al. [19]. However, the meta-analysis of Ndiaye et al. showed a substantially higher prevalence up to 45.8% in OPC [17]. This worldwide overall prevalence is largely influenced by the very high prevalence in North America (60.4%), while the overall prevalence in Europe lands at 41.4%. The differences can probably be explained, in part, by the differences in the geographic origin of the samples, as well as the high heterogeneity in laboratory procedures and assays used in the various studies.

Due to its high sensitivity, HPV DNA PCR-based assays can detect low viral copy numbers, which may not trigger carcinogenesis. These assays cannot distinguish between transcriptionally-active and passenger HPV infections. Therefore, other assays (HPV E6*I mRNA, and p16^(INK4a)^ staining) were evaluated to assess whether combined testing could improve the specificity. Only 28% of the samples with a valid HPV E6*I mRNA result were positive for HPV DNA and p16^(INK4a)^, which might be the fraction of oropharyngeal cancers caused by HPV. After all, the detection of HPV E6 and/or E7 mRNA is seen as the gold standard in this context, because HPV-driven carcinomas critically depend on the continuous expression of E6/E7 oncogenes of hrHPVs [23, 26, 41]. However, the detection of viral transcripts is challenging, not only because the RNA extraction step is laborious and time consuming, but specific infrastructures and equipment are needed. Therefore, HPV RNA assays may not be routinely feasible in all laboratories or on all tissue samples.

HPV-driven OPC represents an increasing health problem, and therefore, reliable and accurate diagnosis becomes essential. In our Belgian HPV-AHEAD study, HPV DNA PCR testing alone was 100% sensitive, but less specific (92.5%) compared to mRNA testing. The inherent strength of the PCR-based methodology lies in its capacity to detect very small amounts of HPV DNA. At the same time, strict laboratory procedures and controls are critical in reducing contamination-related false-positive findings [42]. Immunohistochemistry for p16^(INK4a)^ is most widely used as a surrogate marker for hrHPV infection in FFPE tissues, also for oropharyngeal squamous cell carcinoma. p16^(INK4a)^ IHC testing alone was a bit less sensitive (96.4%), but co-presence of hrHPV DNA and p16^(INK4a)^ positivity was similarly sensitive (96.4%) and more specific (97.0%) compared to each test separately. In literature, several authors have advocated against the use of either p16^(INK4a)^ or hrHPV DNA alone as indicators of HPV-induced etiology in cancers, but recommended their combined use as a reliable and practical approach to differentiate HPV-induced from HPV-unrelated tumours [26, 43–45]. The meta-analysis performed by Prigge et al. [46] also showed a similarly high (pooled) sensitivity of the combined testing (93%) and either p16^(INK4a)^ (94%) or HPV DNA (98%) alone, and the (pooled) specificity (96%) was significantly higher than either testing method alone (83% and 84%, respectively). These accuracy data were confirmed by these Belgian results.

Relative sensitivities and specificities were not significantly different from unity in our study, nor in the meta-analysis of Prigge et al. [46], where significance was only reached for the relative specificity of the combination of HPV DNA PCR and p16^(INK4a)^ tests versus p16^(INK4a)^ IHC [rel. spec.: 1.13 (95% CI 1.04–1.23)], by pooling from multiple studies.

Furthermore, better clinical outcomes have been reported for patients with HPV-induced compared to HPV-unrelated OPC [6–11]. HPV-positive OPC patients show better age-standardised survival than HPV-negative counterparts, leading to investigation of de-intensified therapies to improve their quality of life. However, recent trials have shown worse survival outcomes [47–49]. Therefore, it is crucial to further characterize the molecular mechanisms defining HPV-driven OPC in order to identify novel prognostic markers and, in a more distant future, probable targets for more tailored and effective therapies for this subtype of OPC patients, with a therapeutic success at least equal or improved compared to current treatment regimens.

Improved prognostication by combined p16^(INK4a)^ and hrHPV DNA detection compared to single marker analysis has been demonstrated in a large meta-analysis on tumours in the head and neck region [50]. In our study, 97% (92/95) of the patients would have been correctly diagnosed with the combined testing approach. However, one 58-year old male (current smoker and alcohol consumer) with a T1 (N0 M0) microinvasive tonsillar tumour might have been considered spuriously as having a bad prognosis by applying this combined test strategy, as his tumour would have been classified as a HPV-unrelated case, being p16^(INK4a)^ negative while actually both DNA and mRNA HPV16 positive. More than six years after diagnosis, the patient was alive without any evidence of disease.

On the other hand, disease-specific survival rates are not improved among HPV DNA-positives where HPV is not the cause of carcinogenesis. In our series of 95 Belgian OPC patients, the number of false positive cancers would be reduced from five to two with the combined p16 + DNA detection. The two HPV-unrelated cancer patients were male and diagnosed with a tonsillar cancer. The first patient, with a Tx N2 M1 tonsil NOS tumour, was a heavy drinker and current smoker who only underwent surgery and had a recurrence within 6 months. He died 2 years after being diagnosed. The other patient, with a T3 N2 tonsil NOS tumour, received surgery followed by radiotherapy and concurrent chemotherapy for 2 months. At the last follow-up date, this patient was alive, but with evidence of residual disease. Of note, these two HPV-unrelated patients clearly had a much worse disease status at diagnosis and thereby reduced survival chances compared to the above described HPV-driven tonsillar cancer patient.

Ninety-seven percent of the OPC patients in our study would have been correctly diagnosed as patients with a HPV-driven cancer by combined p16^(INK4a)^ and hrHPV DNA (p16 + DNA) detection. Sequential testing algorithms (p16 → DNA and DNA → p16) resulted in equally accurate results, however, with a 60% reduction in the number of tests needed to be performed. This will cause a substantial reduction in costs and laboratory time, while providing the same clinical value. Especially the algorithm of p16^(INK4a)^ on all samples followed by HPV DNA PCR on p16^(INK4a)^-positive samples only would be a practical strategy. After all, p16^(INK4a)^ IHC can easily be combined with standard histology when a H&E-stained tissue section is prepared for examination by a pathologist. It is a routine diagnostic procedure, at a relatively low cost, available as a validated in-vitro diagnostic reagent and a fully automated protocol, which generates results within several hours after the procedure has been requested. HPV DNA PCR is also a standard laboratory procedure with high throughput and quick results, although against a higher cost. Preselection by p16^(INK4a)^-staining therefore reduces the workload and associated costs, and the combination of HPV DNA and p16^(INK4a)^ testing leads to an important reduction of the number of false-positive observations versus the use of either of these assays alone.

## Limitations

The limited number of OPC cases and the respective anatomical subsites present in this cohort could have influenced the precision of the accuracy. However, more studies will emerge from the HPV-AHEAD consortium and a new updated meta-analysis on diagnostic accuracy of p16^(INK4a)^ immunohistochemistry in oropharyngeal squamous cell carcinomas is planned (which will include all studies from the meta-analysis by Prigge et al. [46], several HPV-AHEAD datasets and studies identified from the literature published after the meta-analysis of Prigge). A fine anatomical sub-classification can be incorporated as covariate, which hopefully yields statistical power.

## Conclusions

In conclusion, combined testing for hrHPV DNA and p16^(INK4a)^ enhances specificity up to 97%, while maintaining high sensitivity (96%), compared to single testing. The diagnostic test combination represents an accurate and accessible testing strategy in the clinical setting for diagnosis of HPV-induced OPC (especially for base of tongue and tonsillar cancers), allowing discriminant prognostication.

## Supplementary Information

**Additional file 1: **hrHPV DNA PCR versus p16INK4a and combined hrHPV DNA/p16INK4a versus p16INK4a and hrHPV DNA PCR, for RNA positive and negative oropharyngeal cancers.

## Data Availability

The datasets used and/or analysed during the current study are available from the corresponding author on reasonable request.

## Abbreviations

CI: Confidence interval
FFPE: Formalin-fixed, paraffin-embedded
H&E: Hematoxylin and eosin
HNC: Head and neck cancers
HPV: Human papillomavirus
hrHPV: High-risk human papillomavirus
IHC: Immunohistochemistry
OPC: Oropharyngeal cancer
p16: p16^(INK4a)^
PCR: Polymerase chain reaction
RNA+: mRNA-positive
S: Section
ubC: Ubiquitin C
