# Supplementary material for: Accuracy of high-risk HPV DNA PCR, p16(INK4a) immunohistochemistry or the combination of both to diagnose HPV-driven oropharyngeal cancer
Source: BMC Infect Dis. 2022 Aug 6;22:676. doi: 10.1186/s12879-022-07654-2 (PMC9357318; doi:10.1186/s12879-022-07654-2)
Supplement: Supplementary file 1 — Additional file 1: hrHPV DNA PCR versus p16INK4a and combined hrHPV DNA/p16INK4a versus p16INK4a and hrHPV DNA PCR, for RNA positive and negative oropharyngeal cancers. [file 12879_2022_7654_MOESM1_ESM.docx]

**Additional file 1: hrHPV DNA PCR versus p16^INK4a^ and combined hrHPV DNA/p16^INK4a^ versus p16^INK4a^ and hrHPV DNA PCR, for RNA positive and negative oropharyngeal cancers**

|  |  |  | **p16** | |  |
| --- | --- | --- | --- | --- | --- |
| **RNA positive oropharynx** | | | Pos | Neg |  |
|  |  |  | **N** | **N** | Total |
| **hrHPV DNA** | | Pos | **27** | **1** | 28 |
|  |  | Neg | **0** | **0** | 0 |
|  |  | Total | **27** | **1** | 28 |
|  |  |  |  |  |  |
| **p16+DNA** |  | Pos | **27** | **0** | 27 |
|  |  | Neg | **0** | **1** | 1 |
|  |  | Total | **27** | **1** | 28 |
|  |  |  | **hrHPV DNA** | |  |
|  |  |  | Pos | Neg |  |
|  |  |  | **N** | **N** |  |
| **p16+DNA** |  | Pos | **27** | **0** | 27 |
|  |  | Neg | **1** | **0** | 1 |
|  |  | Total | **28** | **0** | 28 |
|  |  |  |  |  |  |
|  |  |  | **p16** | |  |
| **RNA negative oropharynx** | | | Pos | Neg |  |
|  |  |  | **N** | **N** | Total |
| **hrHPV DNA** | | Pos | **2** | **3** | 5 |
|  |  | Neg | **3** | **59** | 62 |
|  |  | Total | **5** | **62** | 67 |
|  |  |  |  |  |  |
| **p16+DNA** |  | Pos | **2** | **0** | 2 |
|  |  | Neg | **3** | **62** | 65 |
|  |  | Total | **5** | **62** | 67 |
|  |  |  | **hrHPV DNA** | |  |
|  |  |  | Pos | neg |  |
|  |  |  | **N** | **N** |  |
| **p16+DNA** |  | Pos | **2** | **0** | 2 |
|  |  | Neg | **3** | **62** | 65 |
|  |  | Total | **5** | **62** | 67 |
